# Screening diverse soybean genotypes for drought tolerance by membership function value based on multiple traits and drought-tolerant coefficient of yield

**DOI:** 10.1186/s12870-020-02519-9

**Published:** 2020-07-08

**Authors:** Chunjuan Yan, Shuhong Song, Wenbin Wang, Changling Wang, Haibo Li, Feng Wang, Shengyou Li, Xugang Sun

**Affiliations:** 1 Crop Institute, Liaoning Academy of Agricultural Science, Shenyang, 110161 Liaoning China; 2 School of Agriculture, Jilin University of Agricultural Science & Technology, Jilin, 132101 China; 3 Institute of Agro-environmental Protection, MOA, Tianjin, 300191 China

**Keywords:** Soybean, Screening drought tolerance genotypes, Membership function value, Drought-tolerant coefficient

## Abstract

**Background:**

Drought is a major limiting factor seriously influencing worldwide soybean production and its impact on yield, morphological and physiological traits depend on the timing it occurs and the intensity of water shortage. Only limited research has however been conducted on identifying the drought-tolerant genotypes at different growth stages (vegetative growth phase, reproductive growth phase and the whole growth phase) as well as evaluate the effectiveness and reliability of multiple phenotypic and yield-related characteristics in soybean.

**Results:**

Two pot experiments and a 2-year field experiment were conducted to evaluate soybean drought tolerance at different growth stages. The membership function value of drought tolerance (MFVD) was used to identify drought-resistant cultivars during vegetative growth phase and reproductive growth stage; the relative drought index (RDI) of yield was used to assess drought-resistant cultivars during the whole growing period. In this study, regression models built based on MFVD indicated that the variation of drought tolerant coefficient (DC) of R/S, TRL, LAI and RSR could explain 73.70% of the total variation at vegetative growth phase. However, higher heritability only found in LAI and RSR, indicating the two traits could serve as reliable criteria for drought evaluation. Similarly, the DC of SPP, YPP, PH, PB, MSNN and STB could explain 94.30% of the total variation in MFVD according to stepwise multiple linear regression analyses at reproductive growth phase. Thus, these six traits were identified as indicators for screening drought resistance genotypes in soybean. In addition, correlation analysis revealed that the MFVD was significantly positively correlated with the DC_RB_, DC_R/S_, DC_RSA_, DC_RSR_ and DC_RBR_ at vegetative growth phase and DC_YPP_, DC_SPP_, DC_RB,_ and DC_PB_ at reproductive growth phase. This indicated that these traits were closely related to the drought resistance of plants.

**Conclusions:**

LD24, JD36 and TF31 of vegetative growth phase, and TD37 and LD26 of reproductive growth phase were identified with drought tolerant and highly drought tolerant, respectively. Moreover, 30 accessions with drought tolerance were screened in the field trial and could be applied for the drought resistance of other genotypes by cross-breeding.

## Background

Soybean (*Glycine max* L. Merr.), as an indispensable source of protein, oil and micronutrients in human diets and animal fodders, has become a crucial and economical agricultural crop in the world based on its excellent nutritional value and health benefits [1]. However, drought stress, which is the most important abiotic restriction, can have devastating effects on the stability and productivity of soybean in many semi- and arid areas of the world [2, 3]. It has been extensively reported that drought stress can directly induce a wide range of injury symptoms in plants, such as the inhibition of plant photosynthesis [4, 5], increased oxidative [6], and changes in metabolism [7]. Furthermore, drought stress led to the decrease in leaf area, pod yield, plant height, 1000-seed weight, harvest index, seed yield, etc. [8]. It was estimated that about 40% soybean yield decrease was caused by drought stress [9]. Depending on hybrid characteristics, soybeans use about 450–700 mm of rain fall during the growing season [10]. Improving the drought resistance of varieties is thus a key measure for reducing yield losses and stabilizing crop production under drought condition. The effects of drought stress on plant depend not only on the characteristics (duration, intensity) of the stress but also on the timing of occurrence relative to the development cycle of the plant. The most critical period for water stress in soybean was the flowering stage and the period following flowering [11]. The yield formation was sensitive when mild water deficit happened during the seedling phase, and became more sensitive to serious water deficit occurring at the flowering-podding phase in soybean [8]. Therefore, it is necessary to evaluate the response of soybean with different genetic germplasms to drought stress at diverse growth stages.

Drought tolerance is defined as the relative yield of a genotype compared to other genotypes subjected to the same drought stress [12]. Although yield was the primary characteristic for measurement of drought resistance on water deficit condition in many crops [13–15], the secondary characteristic might be specifically appropriate to improve selection response to drought stress condition. Relative water content, chlorophyll content, and ascorbic acid could be used as secondary indicators for selecting drought-tolerant genotypes [16, 17]. In this study, a plant is said to have drought tolerance if it can maintain better phenotypic traits and achieve higher yields under drought conditions. Drought-tolerant coefficient (DC), supply a measure of drought effects based on the reduction of each trait under water stress conditions in comparison to well-watered conditions, and therefore used for identification of drought-tolerant genotypes [18–20]. The membership function value of drought tolerance (MFVD) calculated from DC provided a comprehensive evaluation method for drought resistance of materials based on multi-indicator determination [21, 22]. According to this method for drought resistance assessment, several studies have reported that some physiological traits such as photosynthetic rate, leaf chlorophyll content, superoxide dismutase (SOD) activity and some yield-related traits such as spikelet number, grain number per spike, grain yield per plant were affected by soil water stress, and have been considered as evaluation parameters of drought resistance in other crops [23–26]. The direct impact on soybean that occurs due to drought stress are the decrease in yield and its component such as number of pods, number of seeds and seed weight [27]. Soybean plants subjected to water stress during flowering and vegetative growth stages had significantly lower total dry matter and yields [28]. Therefore, it is essential to carry out drought tolerance evaluation of soybean phenotypic and yield-related traits.

Plant root architecture has been also reported to be associated with water stress tolerance in various crops [29, 30]. The ability of a plant to modify its root distribution to exploit deeper stored soil water may be an important mechanism to avoid drought [31]. Deep rooting, root length density and root distribution have been identified as drought adaptive traits [32, 33] which can be used as selection criteria for drought resistance in other crops. However, the assessment on some important morphological and physiological traits, such as root length, root area and leaf area index (LAI) of soybean lines under different water regimes to examine thoroughly how soybean genotypes respond to drought in terms of these traits has not been clearly demonstrated.

The present investigation was carried out to identify the suitable soybean genotypes for drought tolerance at different growth stages combining with the value of membership function (MFVD) and drought-tolerant coefficient (DC). Moreover, this study also aimed to evaluate the use of multiple phenotypic and yield-related traits as secondary indices for drought resistance assessment.

## Results

### Response of traits measured and calculated to water stress

#### Assessing drought tolerance at vegetative growth stage (Expt. 1)

The variation was confirmed by the average value, standard deviation (SD) and the drought-tolerant coefficient (DC) of each trait when water was controlled at vegetative growth phase (VGP) were presented in the Table 1. In response to water-stressed (WS) regime, the mean value of 7 traits decreased but the others increased compared with that in the well-watered (WW) regime. Leaf area index (LAI) showed the lowest DC (0.59) which declined by 41.16%, indicating that LAI was the most sensitive trait to water stress in this group of genotypes. In addition, decreased in WS conditions the mean values of plant height (PH), shoot biomass (SB), root biomass (RB), total root length (TRL), root surface area (RSA) and root volume (RV) by 20.13, 38.53, 5.82, 13.41, 14.58 and 9.94%, respectively, but increased those of root/shoot ratio (R/S), root average diameter (RD), root length ratio (RLR), root surface area ratio (RSR), root volume ratio (RVR), root average diameter ratio (RDR), root biomass ratio (RBR) by 50.00, 14.55, 25.89, 22.49, 25.14, 83.33, and 38.09%, respectively, as compared with the WW regime (Table 1). This indicated that the adverse effects of drought stress on shoots were greater than roots. Furthermore, there was significant difference between the DC of RDR and DC of other traits according to Duncan’s multiple comparisons (*P* < 0.05).

**Table 1:** Means value, standard deviation (SD) of traits under well-watered (WW) and water-stressed (WS) regimes and the drought-tolerant coefficient (DC) of each trait at vegetative growth stage

Traits	Mean ± SD (WW)	Mean ± SD (WS)	Mean ± SD (DC)
PH (cm)	45.05 ± 1.78	35.98 ± 1.93	0.80 ± 0.01 ef
LAI	3.11 ± 0.44	1.83 ± 0.13	0.59 ± 0.05 f
**SB (g plant**^**−1**^**)**	7.06 ± 1.03	4.34 ± 0.25	0.62 ± 0.06 f
**RB (g plant**^**−1**^**)**	1.89 ± 0.18	1.78 ± 0.04	0.95 ± 0.07 de
R/S	0.28 ± 0.02	0.42 ± 0.01	1.53 ± 0.04 b
TRL (m)	51.15 ± 1.81	44.29 ± 2.37	0.87 ± 0.02 e
RSA (cm^2^)	969.69 ± 62.85	828.29 ± 61.42	0.86 ± 0.10 e
RV (cm^3^)	14.69 ± 0.83	13.23 ± 1.10	0.91 ± 0.12 e
RD (mm)	0.55 ± 0.02	0.63 ± 0.04	1.15 ± 0.11 cd
RLR (m/g)	5.91 ± 0.55	7.44 ± 0.15	1.38 ± 0.19 c
RSR (cm^2^/g)	113.21 ± 8.43	138.67 ± 8.38	1.23 ± 0.05 c
RVR (cm^3^/g)	1.75 ± 0.35	2.19 ± 0.18	1.29 ± 0.36 bc
RDR (mm/g)	0.06 ± 0.01	0.11 ± 0.01	1.87 ± 0.07 a
RBR	0.21 ± 0.01	0.29 ± 0.01	1.37 ± 0.03 bc

Means followed by the same letter within the third column are not significantly different (*p* ≤ 0.05) as determined by Duncan’s multiple-range test (*n* = 3)

#### Assessing drought tolerance at the reproductive growth stage (Expt. 2)

Phenotypic variation was confirmed by the average phenotypic value, SD and the DC of each trait when water was controlled at reproductive growth phase (RGP) (Table 2)**.** The mean value of 8 traits decreased, while only one trait increased under water-stressed regime. Root biomass (RB) showed the largest drought coefficient (1.01) which increased by 2.22%, demonstrating that this trait was the less impacted by drought conditions in this group of cultivars. The mean values of hundred seeds weight (HSW) and main stem node number (MSNN) were reduced in WS conditions by 0.16 and 4.83%, respectively. And there was no significant difference between the drought-tolerant coefficient (DC) of the two and the DC of RB according to Duncan’s multiple-range test, indicating that the three agronomic traits were less affected by soil moisture. Moreover, for these varieties, WS treatment decreased the mean values of plant height (PH), pods per plant (PPP), seeds per plant (SPP), yield per plant (YPP), stem biomass (STB), pod biomass (PB) by 8.49, 19.09, 25.05%, 24.24.80, 26.23, and 20.98%, respectively, as compared with WW treatment.

**Table 2:** Means value, standard deviation (SD) of traits under well-watered (WW) and water-stressed (WS) regimes and the drought-tolerant coefficient (DC) of each trait at reproductive growth phase

Traits	Mean ± SD (WW)	Mean ± SD (WS)	Mean ± SD (DC)
PH (cm)	96.35 ± 11.70	88.81 ± 9.40	0.92 ± 0.02 abc
MSNN	15.54 ± 1.31	15.11 ± 0.92	0.97 ± 0.02 ab
PPP	32.94 ± 13.51	27.66 ± 6.00	0.88 ± 0.18 abc
SPP	67.65 ± 28.18	54.10 ± 18.41	0.82 ± 0.10 bc
HSW (g)	19.35 ± 2.67	19.32 ± 2.18	0.99 ± 0.03 a
YPP **(g plant**^**−1**^**)**	12.61 ± 4.71	10.15 ± 3.24	0.82 ± 0.04 c
RB **(g plant**^**− 1**^**)**	3.61 ± 0.58	3.69 ± 1.03	1.01 ± 0.12 a
STB **(g plant**^**−1**^**)**	9.72 ± 2.71	7.70 ± 1.97	0.79 ± 0.04 c
PB **(g plant**^**−1**^**)**	4.67 ± 1.84	3.86 ± 1.29	0.84 ± 0.06 bc

Means followed by the same letter within the third column are not significantly different (*p* ≤ 0.05) as determined by Duncan’s multiple-range test (*n* = 3)

### Relationship of the same trait between WS and WW treatments

**Fig. 1:**
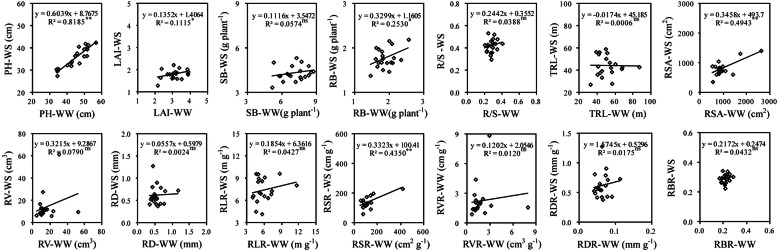
Relationship of the same trait between WS and WW treatments at vegetative growth phase. ns r is no significance; * r is significance at level of 0.05; ** r is significance at levels of 0.01. The same as below

**Fig. 2:**
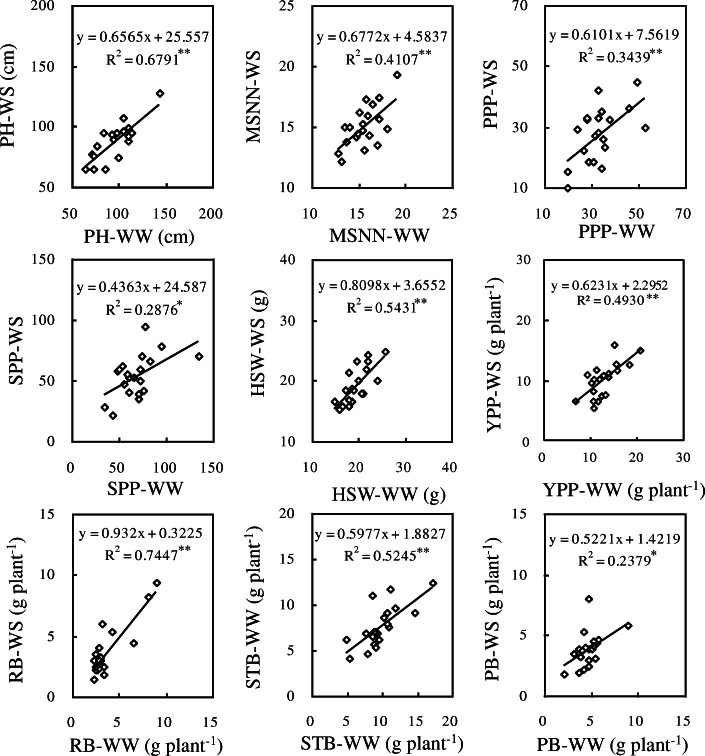
Relationship of the same trait between WS and WW treatments at reproductive growth phase

**Fig. 3:**
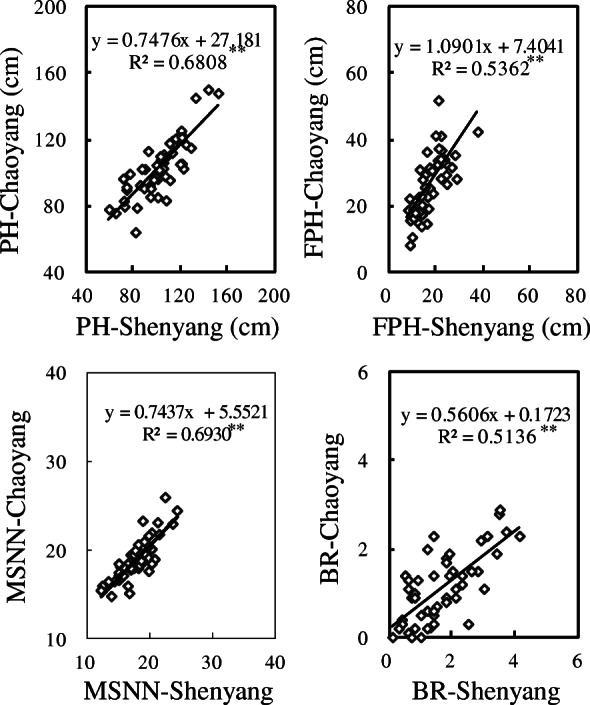
Relationship of the same trait between well-watered condition (Shenyang) and water stress condition (Chaoyang)

Significant and positive correlations were observed between WS and WW regimes for the same traits including PH (*r*^*2*^ = 0.819, *P* < 0.01), LAI (*r*^*2*^ = 0.192, *P* < 0.05), RB (*r*^*2*^ = 0.253, *P* < 0.05), RSA (*r*^*2*^ = 0.494, *P* < 0.01) and RSR (*r*^*2*^ = 0.435, *P* < 0.01) when water was controlled at vegetative growth phase (Fig. 1), and thus the above five parameters could serve as reliable characters in drought screening. At reproductive growth stage, correlations between WW and WS regimes for the same indicators in all the investigated traits (PH, *r*^*2*^ = 0.679, *P* < 0.01; MSNN, *r*^*2*^ = 0.411, *P* < 0.01; PPP, *r*^*2*^ = 0.344, *P* < 0.01; SPP, *r*^*2*^ = 0.288, *P* < 0.05; HSW, *r*^*2*^ = 0.543, *P* < 0.01; YPP, *r*^*2*^ = 0.493, *P* < 0.01; RB, *r*^*2*^ = 0.745, *P* < 0.01; STB, *r*^*2*^ = 0.525, *P* < 0.01; PB, *r*^*2*^ = 0.238, *P* < 0.05) were all significant (Fig. 2). At the whole growth stage, it was also found that the same indicators of all investigated agronomic traits (PH, *r*^*2*^ = 0.681, *P* < 0.01; FPH, *r*^*2*^ = 0.536, *P* < 0.01; MSNN, *r*^*2*^ = 0.693, *P* < 0.01; BR, *r*^*2*^ = 0.514, *P* < 0.01) of Shenyang were positively correlated with those of Chaoyang (Fig. 3).

### Genetic variation and broad sense heritability of the investigated traits

#### Assessing drought tolerance at vegetative growth stage (Expt. 1)

The ANOVA revealed that there were significant effects (*P* < 0.05) for 9 traits including plant height (PH), leaf area index (LAI), shoot biomass (SB), root/shoot ratio (R/S), root surface area (RSA), root length ratio (RLR), root surface area ratio (RSR), root average diameter ratio (RDR), root biomass ratio (RBR) between two water regimes and 11 tested traits including PH, LAI, SB, total root length (TRL), root surface area (RSA), root volume (RV), root average diameter (RD), root length ratio (RLR), root surface area ratio (RSR), root volume ratio (RVR), root average diameter ratio (RDR) among 20 cultivars (Table 3). Only 5 traits (TRL, RV, RD, RVR, RDR) varied greatly (*P* < 0.05) between variety and water regime interactions. The genetic variation coefficient (*CVg)* ranged from 16.49 to 38.95% on WW regime and from 15.32 to 41.62% on WS regime for the 14 traits, respectively (Table 3). The lowest broad sense heritability (*H*^*2*^*)* was estimated for R/S (0.34), suggesting that the soil moisture made a larger contribution to the variation of the trait. The higher *H*^*2*^ estimates, in these traits were obtained for PH, LAI, RB, RSA and RSR with a significant linear relationship between WS and WW regimes (Fig. 1 and Table 3).

**Table 3:** Analysis of variance (ANOVA), genetic variation coefficient (*CVg*) and broad sense heritability (*H*^*2*^) of each trait on two water conditions of 20 varieties at vegetative growth phase

Variation Source	df	Means of squares													
		PH (cm)	LAI	SB	RB	R/S	TRL	RSA	RV	RD	RLR	RSR	RVR	RDR	RBR
Replication (R)	**2**	138.04	3.11	16.09	0.47	0.01	638.84	80,066.06	298.45	0.08	0.16	7099.86	6.40	0.00	0.00
Water (W)	**1**	2470.67^***^	49.59^*^	223.29^*^	0.34	0.63^***^	1410.16	589,699.18^*^	18.64	0.17	77.34^*^	16,762.72^*^	6.77	0.05^*^	0.19^***^
Variety (V)	19	186.08^***^	0.58^**^	2.48^**^	0.25	0.01	358.16^**^	657,814.55^***^	500.60^***^	0.12^***^	10.28^**^	16,765.40^***^	9.18^***^	0.00^***^	0.00
**W × V**	19	16.67	0.36	1.71	0.11	0.01	374.67^**^	187,211.60	282.48^**^	0.11^***^	6.78	5149.34	7.38^***^	0.00^***^	0.00
**Error**	**38**	12.09	0.26	1.13	0.15	0.01	157.98	112,709.22	108.32	0.03	4.35	3403.59	2.51	0.00	0.00
***CV***_***g***_**(%) (WW)**		16.49	25.51	23.06	23.30	27.66	27.69	30.36	38.95	36.08	34.84	38.24	38.77	34.08	21.04
***CV***_***g***_**(%) (WS)**		15.32	21.31	22.00	20.92	23.32	27.75	30.74	38.08	37.02	31.89	32.29	41.62	39.82	16.36
*H*^*2*^		**0.91**	0.62	0.44	0.63	0.34	0.35	0.74	0.58	0.45	0.47	0.72	0.47	0.43	0.36

*,**,*** Mean of squares significant at *p* < 0.05, 0.01, and 0.001, respectively

#### Assessing drought tolerance at the reproductive growth stage (Expt. 2)

Analysis of variance, genetic variation coefficient (*CVg*) and broad sense heritability (*H*^*2*^) of each trait under WS and WW regimes at reproductive growth phase were given in Table 4. Extremely significant differences (*P* < 0.01) in plant height (PH), main stem node number (MSNN), pods per plant (PPP), seeds per plant (SPP), hundred seeds weight (HSW), yield per plant (YPP), root biomass (RB), stem biomass (STB), and pod biomass (PB) occurred between the tested varieties. The great differences were also observed in PH, MSNN, SPP, HSW, STB and PB between the interactions. The *CVg* ranged from 13.29 to 47.99% under WW condition and from 12.87 to 50.30% under WS condition for 9 traits, respectively. The maximum and minimum broad sense heritability (*H*^*2*^) was estimated for PH (0.90) and PB (0.71), respectively. The *H*^*2*^ values for the 9 investigated traits were all more than 0.71, for instance, MSNN (0.81), SPP (0.73), etc. This indicated that the variations of the traits were mostly due to genetic differences and these traits were highly heritable.

**Table 4:** ANOVA, *CVg* and *H*^*2*^ of each trait on two water conditions of 20 varieties at reproductive growth phase

Variation Source	df	Means of squares								
		PH (cm)	MSNN	PPP	SPP	HSW	YPP	RB	STB	PB
Replication (R)	**2**	4449.43	49.58	4184.43	21,640.29	234.28	632.88	26.02	217.40	98.10
Water (W)	**1**	1706.30^*^	5.63	837.41^*^	5504.69*	0.02	181.11*	0.19	123.54*	19.62*
Variety (V)	**19**	1669.39^***^	14.17^***^	368.50^***^	1717.99^***^	41.92^***^	46.92^***^	22.44^***^	32.44^***^	8.51^***^
**W × V**	**19**	182.18^***^	3.11^***^	96.20	534.98^*^	6.44^**^	8.37	1.68	5.48^*^	2.94^**^
**Error**	**38**	43.72	0.58	90.16	293.81	2.94	9.82	1.38	2.68	1.30
***CV***_***g***_**(%) (WW)**		22.89	13.29	47.88	51.73	19.67	46.00	16.06	38.62	47.99
***CV***_***g***_**(%) (WS)**		20.10	12.87	47.71	47.04	18.63	42.93	27.93	39.34	50.30
*H*^*2*^		0.90	0.81	0.74	0.73	0.86	0.82	0.88	0.84	0.71

*,**,*** Mean of squares significant at *p* < 0.05, 0.01, and 0.001, respectively

#### Assessing drought tolerance at the whole growth stage (Expt. 3)

Variety (V), location (L) and L × V interactions had significant effects (*P* ≤ 0.05) for yield, plant height (PH), first pod height (FPH), branches (BR) in 2 years (Table 5). The main effects associated with variety and L × V interaction term were also significant (*P* ≤ 0.05) with respect to main stem node number (MSNN) in both years. However, location did not significantly affect MSNN in 2014, suggesting that MSNN was less affected by the different rainfall conditions. The *CVg* for the 5 investigated traits ranged from 13.77 to 72.83% in Shenyang and from 15.72 to 88.00% in Chaoyang during 2014–2015, respectively (Table 5). In addition, *CVg* values of 3 traits, i.e., yield, PH, MSNN ranged between 10 and 30%, and for first pod height (FPH) and branches (BR), *CVg* values were more than 30% in 2014–2015. Higher *H*^*2*^ was observed for the five traits tested in the 2 years, indicating that the phenotypic variations of these traits in this group of genotypes were mostly due to genetic differences and they were highly heritable traits.

**Table 5:** ANOVA, *CVg* and *H*^*2*^ of each trait under well-watered condition (Shenyang) and water stress condition (Chaoyang) of 50 varieties in both years

Variation Source	df	Means of squares									
		2014					2015				
		Yield (kg ha^−1^)	PH (cm)	FPH (cm)	MSNN	BR	Yield (kg ha^−1^)	PH (cm)	FPH (cm)	MSNN	BR
Replication (R)	**2**	94,924.81	181.05	47.54	5.29	0.36	1646.295	161.05	141.67	0.06	0.07
Location (L)	**1**	8,300,616.00^*^	4901.29^*^	4434.42^*^	103.57	6.57^*^	9372584^*^	1889.55^*^	7868.95^*^	44.96^*^	47.88^*^
Variety (V)	**49**	880,734.50^***^	1979.29^***^	414.44^***^	42.99^***^	4.53^***^	46.92^***^	2170.80^***^	293.17^***^	32.11^***^	6.07^***^
L **× V**	**49**	269,710.70^***^	235.37^***^	116.45^***^	5.51^***^	0.97^***^	1,287,688.00^***^	451.74^***^	93.81^***^	6.07^***^	1.64^***^
**Error**	96	94,624.68	10.14	7.05	1.58	0.27	318,273.50	7.52	7.95	0.66	0.22
***CV***_***g***_**(%) (Shenyang)**		13.77	20.54	43.09	18.07	72.52	19.95	16.06	41.48	15.73	72.83
***CV***_***g***_**(%) (Chaoyang)**		21.32	15.72	43.11	14.28	88.00	22.65	27.93	36.19	12.69	83.64
*H*^*2*^		0.74	0.89	0.78	0.88	0.81	0.83	0.93	0.75	0.84	0.78

*,**,*** Mean of squares significant at *p* < 0.05, 0.01, and 0.001, respectively

### Identification and classification of drought tolerance among diverse genotypes

#### Assessing drought tolerance at vegetative growth stage and reproductive growth stage (Expts. 1 and 2)

The MFVD can be used as a comprehensive index to evaluate the drought tolerance of soybean genotypes according to the 14 characteristics during the vegetative growth period and the 9 traits for the reproductive growth period (Table 6). Among the 20 soybean accessions, 3 accessions (LD24, JD36 and TF31) showed drought tolerance (Level 2) when water was controlled at vegetative growth phase, 2 accessions (TD37 and LD26) showed highly drought tolerance (Level 1) water controlled at reproductive growth stage. Thus, these accessions could be used as resources for drought tolerance improvement in soybean breeding. Besides, 3 accessions (LD10, FD17 and LD21) and 1 accession (LD17) was screened for susceptible (Level 4) to water stress during vegetative growth phase and reproductive growth phase, respectively.

**Table 6:** Subordinate function values and class to drought resistance on genotypes tested when water was controlled at vegetative growth phase and reproductive growth phase

Cultivar Name	LD10	LD15	LD17	LD18	LD21	LD23	LD24	LD26	JD36	JD37	KY11	KY12	TF29	TF31	TD37	TD40	TD49	DD12	SN10	FD17
MFVD of VGP	0.33	0.36	0.47	0.40	0.33	0.37	0.52	0.38	0.53	0.43	0.39	0.41	0.40	0.49	0.37	0.45	0.40	0.35	0.41	0.33
Level	4	3	3	3	4	3	2	3	2	3	3	3	3	2	3	3	3	3	3	4
MFVD of RGP	0.48	0.51	0.43	0.55	0.52	0.51	0.45	0.61	0.53	0.49	0.54	0.47	0.51	0.44	0.61	0.45	0.49	0.53	0.52	0.53
Level	3	3	4	3	3	3	3	1	3	3	3	3	3	3	1	3	3	3	3	3

#### Assessing drought tolerance at the whole growth stage (Expt. 3)

**Fig. 4:**
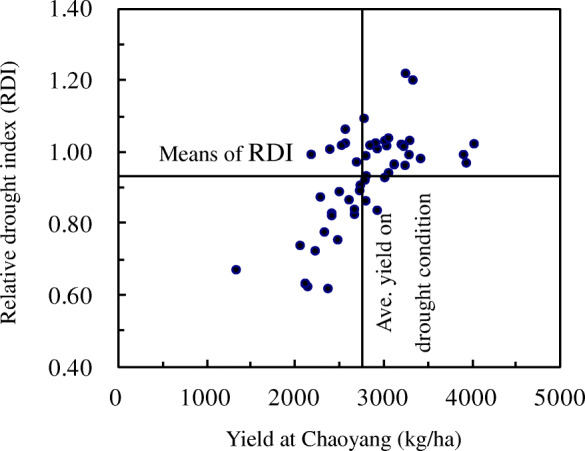
Identification and classification to drought resistance on genotypes tested of the whole growth phase

Fifty soybean genotypes were classified into four groups based on relative drought index (RDI) of yield and yield in semi-arid region in the field experiments during the 2 years (Fig. 4 and Table 7). Group I was comprised of the drought tolerant and high yield genotypes with higher RDI values than average (0.93) of 50 genotypes and higher yield than the average (2785.95 kg ha^− 1^). This group had 23 cultivars including LD24, JD36 and TF31 that have been screened with drought tolerance at vegetative growth phase. The group II comprised of non-drought tolerant and high yield genotypes which produced more than average yield of 50 genotypes at semi-arid zone, but the response to drought stress was lower than the average. There were only 2 genotypes within group II. The group III involved drought tolerance and low yield cultivars produced higher than average soybean yield, but the resistance to drought stress was above the average. There were 7 cultivars in group III including TD37 and LD26 screened with highly drought tolerance when water was controlled at reproductive growth phase. The group IV consisted of non-drought tolerance and low yield accessions including 18 genotypes that showed lower yield and RDI values than the average.

**Table 7:** Identification and classification to drought resistance on genotypes tested of the whole growth stage

Drought tolerance and high yield	Non-drought tolerance and high yield	Drought tolerance and low yield	Non-drought tolerance and low yield
DD14, JD36, JD37, LD18, LD24, LD25, LD29, LD30, SN12, SN16, SN17, TF31, TD39, TD43, TD45, TD46, TD48, TD49, TD50, TD55, TD56, TD57, YW9.	LX2, TD47.	DD11, KY11, LD17, LD23, LD26, LD31, TD37.	DD12, DD13, DD15, FD17, KY12, L08–28, LD15, LD21, LD22, LD28, SN8, SN10, SN11, TD38, TD40, TD42, XY11, YW6.

### Correlation between MFVD and DC of each trait

#### Assessing drought tolerance at vegetative growth stage (Expt. 1)

Correlation coefficients between the membership function value of drought tolerance (MFVD) and the drought-tolerant coefficient (DC) of each trait at vegetative growth stage were analyzed (Table 8). The DC_RB_, DC_R/S_, DC_RSA_, DC_RLR_, DC_RSR_ and DC_RBR_ showed highly positive correlations with the MFVD (Table 8; *P* < 0.05). Moreover, correlation coefficients between the DC of each trait and those of others were also analyzed and shown in Table 8. The highest correlation of DC was found between RBR and R/S (*r* = 1.00; *P* < 0.01), meaning that RBR was closely related to R/S. In addition, DC_PH_, DC_LAI_ and DC_SB_ were also positively correlated with each other. In contrast, a negative correlation occurred between DC_R/S_ and DC_LAI_, DC_SB_.

**Table 8:** Correlation coefficients between the membership function value of drought tolerance (MFVD) and the drought-tolerant coefficient (DC) of each trait at vegetative growth stage

	MFVD	DC_PH_	DC_LAI_	DC_SB_	DC_RB_	DC_R/S_	DC_TRL_	DC_RSA_	DC_RV_	DC_RD_	DC_RLR_	DC_RSR_	DC_RVR_	DC_RDR_
DC_PH_	− 0.14													
DC_LAI_	−0.22	0.75^**^												
DC_SB_	−0.10	0.83^**^	0.89^**^											
DC_RB_	0.47^*^	0.31	0.24	0.18										
DC_R/S_	0.66^**^	− 0.40	− 0.49^*^	− 0.63^**^	0.62^**^									
DC_TRL_	0.40	0.35	0.11	0.15	0.41	0.21								
DC_RSA_	0.52^*^	0.14	− 0.03	− 0.10	0.38	0.43	0.56^**^							
DC_RV_	−0.35	− 0.10	− 0.03	0.05	− 0.06	− 0.42	− 0.08	− 0.14						
DC_RD_	0.17	0.28	0.23	0.23	0.48*	0.21	0.07	0.17	0.10					
DC_RLR_	0.47^*^	0.00	−0.28	−0.28	0.19	0.40	0.87^**^	0.65^**^	−0.07	−0.08				
DC_RSR_	0.53^*^	−0.14	− 0.32	−0.41	0.22	0.56^**^	0.48^*^	0.93^**^	−0.10	0.13	0.73^**^			
DC_RVR_	−0.26	− 0.29	−0.24	− 0.20	−0.13	− 0.28	−0.15	− 0.16	0.94^**^	− 0.11	−0.01	− 0.42		
DC_RDR_	0.25	−0.01	−0.05	− 0.09	0.35	0.39	−0.04	0.27	−0.13	0.92^**^	0.00	0.34	−0.16	
DC_RBR_	0.65^**^	−0.45^*^	−0.52^*^	− 0.67^**^	0.59^**^	1.00^**^	0.19	0.43^*^	−0.13	0.21	0.41	0.58^**^	−0.27	0.40

* r is significance at level of 0.05; ** r is significance at level of 0.01; *** r is significance at level of 0.001

#### Assessing drought tolerance at the reproductive growth stage (Expt. 2)

Correlation coefficients between the MFVD and DC of each trait at reproductive growth stage were listed in Table 9. The great positive correlation occurred between MFVD and DC_SPP_ (*r* = 0.47; *P* < 0.05), DC_YPP_ (*r* = 0.42; *P* < 0.05), DC_PB_ (*r* = 0.41; *P* < 0.05) and DC_RB_ (*r* = 0.46; *P* < 0.05). Besides, correlation coefficients between the DC of each trait were available in Table 9 when water was controlled at reproductive growth stage. Significant positive correlations of DC of tested traits were observed between DC_YPP_ and DC_PH_, DC_YPP_ and DC_MSNN_, DC_YPP_ and DC_PPP_, DC_YPP_ and DC_SPP_, DC_YPP_ and DC_STB_, and DC_YPP_ and DC_PB_, revealing that these traits were closely related to yield per plant in soybean. The significant correlations also occurred between DC_PH_ and DC_MSNN_ (*r* = 0.63; *P* < 0.01), DC_PPP_ and DC_SPP_ (*r* = 0.96; *P* < 0.01), DC_PPP_ and DC_PB_ (*r* = 0.91; *P* < 0.01) (Table 9).

**Table 9:** Correlation coefficients between the MFVD and the DC of each trait at reproductive growth stage

	MFVD	DC_PH_	DC_MSNN_	DC_PPP_	DC_SPP_	DC_HSW_	DC_YPP_	DC_RB_	DC_STB_
DC_PH_	0.22								
DC_MSNN_	0.05	0.63^**^							
DC_PPP_	0.03	0.49^*^	0.57^**^						
DC_SPP_	0.47*	0.55^**^	0.61^**^	0.96^**^					
DC_HSW_	0.15	−0.38	−0.28	−0.64^**^	−0.62^**^				
DC_YPP_	0.42^*^	0.56^**^	0.61^**^	0.84^**^	0.93^**^	−0.36			
DC_RB_	0.46^*^	−0.09	0.00	−0.15	−0.19	− 0.25	−0.32		
DC_STB_	0.21	0.66^**^	0.33	0.47^*^	0.56^**^	−0.49^*^	0.50^*^	0.05	
DC_PB_	0.41^*^	0.44^*^	0.51^*^	0.91^**^	0.87^**^	−0.53^*^	0.84^**^	−0.15	0.43^*^

* r is significance at level of 0.05; ** r is significance at level of 0.01; *** r is significance at level of 0.001

### Drought tolerance explained by multiple DC of traits

#### Assessing drought tolerance at vegetative growth stage (Expt. 1)

Multiple linear stepwise regression to explain the MFVD prediction with the accepted 4 limiting DC of traits at vegetative growth phase were estimated and listed in Table 10. The results showed that the MFVD variation was explained 36.60, 21.00, 10.20, and 5.90% by DC of R/S, TRL, LAI and RSR, respectively. As a result, 73.70% of the variation in MFVD was caused by these DC of 4 traits. Regression coefficient, standard error, *T*-value and probability of the 4 accepted variables at vegetative growth phase were calculated and displayed in Table 11. The prediction equation for MFVD with accepted 4 limiting DC of traits was as:

$$ {Y}_{MFVDV}=0.279-0.176A1+0.110A2+0.082A3+0.024A4 $$

where *Y*_*MFVDV*_ was the membership functions value of drought tolerance for one cultivar at VGP, *A1*, *A2*, *A3* and *A4* were the DC value for R/S, TRL, LAI and RSR, respectively.

**Table 10:** Multiple linear stepwise regression to explain the MFVD with DC of each trait at vegetative growth phase and reproductive growth phase

Variables	Step	Variable entered	Partial *R*^*2*^	Model *R*^*2*^	Sig.
**MFVD** **(**VGP**)**	1	DC_R/S_(A1)	0.366	0.366	***
	2	DC _TRL_ (A2)	0.210	0.776	*
	3	DC _LAI_ (A3)	0.102	0.838	**
	4	DC _RBR_ (A4)	0.059	0.868	*
**MFVD** **(**RGP**)**	1	DC_SPP_ (B1)	0.446	0.902	***
	2	DC_YPP_ (B2)	0.201	0.999	**
	3	DC_PH_(B3)	0.116	0.902	***
	4	DC_PB_ (B4)	0.066	0.953	*
	5	DC_MSNN_ (B5)	0.067	0.987	***
	6	DC_STB_ (B6)	0.047	0.969	*

*MFVD (VGP)* The membership function value of drought tolerance of vegetative growth phase, *MFVD (RGP)* The membership function value of drought tolerance of reproductive growth phase *,**,*** Significant at p < 0.05, 0.01, and 0.001, respectively

**Table 11:** Regression coefficient, standard error, *T*-value and probability of the accepted DC of each trait that can be used to predict the MFVD based on the stepwise regression analysis at vegetative growth phase and reproductive growth phase

Variables	Variable entered	Regression coefficients	Standard error	*T*	Sig.
**MFVD** **(**VGP**)**	Constant	0.280	0.054	5.150	***
	DC_R/S_ (*A1*)	−0.176	0.059	−2.960	*
	DC _TRL_(*A2*)	0.110	0.020	5.400	***
	DC_LAI_ (*A3*)	0.082	0.019	4.310	***
	DC_RBR_ (*A4*)	−0.024	0.014	−1.770	*
**MFVD** **(**RGP**)**	Constant	−0.421	0.014	−30.890	***
	DC_SPP_ (*B1*)	0.165	0.008	21.390	***
	DC_YPP_ (*B2*)	0.108	0.006	18.650	***
	DC_PH_ (*B3*)	0.048	0.005	9.800	***
	DC_PB_ (*B4*)	0.060	0.004	13.670	***
	DC_MSNN_ (*B5*)	0.174	0.009	18.560	***
	DC_STB_ (*B6*)	0.130	0.004	30.500	***

*,**,*** Significant at *p* < 0.05, 0.01, and 0.001, respectively

#### Assessing drought tolerance at reproductive growth stage (Expt. 2)

Multiple linear stepwise regression to explain the MFVD prediction with the accepted 6 DC of traits at reproductive growth phase were estimated and listed in Table 10. The results showed that the MFVD variation was explained 44.60, 20.10, 11.60, 6.60, 6.70, and 4.70% by DC of SPP, YPP, PH, PB, MSNN and STB, respectively. In total, 94.30% of the variation in MFVD was ascribed to these 6 DC of traits. Regression coefficients for the 6 accepted variables were calculated and shown in Table 11. The prediction equation for MFVD with 6 limiting DC of traits was as:

$$ {Y}_{MFVDR}=-0.421+0.165B1+0.108B2+0.048B3+0.060B4+0.174B5+0.130B6 $$

where *Y*_*MFVDR*_ was the membership functions value of drought tolerance for one cultivar at RGP, *B1*, *B2*, *B3*, *B4*, *B5* and *B6* were the DC value for SPP, YPP, PH, PB, MSNN and STB, respectively.

## Discussion

Soybean is one of the major and wide spread crops in the world and is rather sensitive to water stress**.** Many drought-tolerant varieties have been developed for other crops [17, 20, 34, 35]. However, study on a set of accurate, stable, simple and systematic method and index system for identification and selection of drought tolerance in soybean is still limited. It is an important step to improve the identification of soybean drought-tolerant germplasm by screening a number of key and stable traits with high heritability for the identification of drought tolerance in soybean.

Drought stress caused reductions in plant height, pods per plant, 100-kernel weight, yield per plant, LAI, biological yield, root length, root volume, etc. [4, 36–39]. Soybean yield is mainly a function of number of plants, dry matter production, seed numbers and seed size. Water stress have been reported to reduce seed weight, total biomass, pods per plant, seeds per plant, seeds per pod, 100-grain weight, and ultimately caused a decline in soybean yield [40–42]. In this study, some drought traits measured among 20 soybean accessions under WS regimes also showed remarkable decline in PH, LAI, SB, RB, TRL, RSA, RD and RV water controlled at vegetative growth phase. In addition, decline in PH, PPP, SPP, YPP, STB and PB was observed when water was controlled at reproductive growth phase. In contrast, striking increase occurred in R/S, RD, RLR, RSR, RVR, RDR and RBR under water stress condition, as compared with the well water treatment. This showed that drought stress enhanced the proportion of root distribution throughout the plant [43, 44].

The present study from Expt.2 and Expt.3 also showed that all the investigated agronomic traits in the WW treatments were positively correlated with those of the WS treatment. The similar results were obtained by Liu et al. [45]. However, in Expt.1 of this study, only the five traits, i.e., PH, LAI, SB, RSA and RSR had high and positive correlations (*P* < 0.05) between WW and WS conditions. This could be attributed to the previous experimental conditions where water stress was weaker than the present study. In addition, the differences in the tested crops and the measured indicators were also the main reason.

The significant differences in variety and water stress were observed on plant height, LAI, shoot biomass, root biomass, R/S, total root length, etc. [46–49]. The ANOVA analysis in Expt.1 also indicated that there were significant differences for some traits including PH, LAI, SB, RSA, RLR, RSR, etc. between among 20 cultivars and two water regimes. The genetic variation for the trait under selection and a higher heritability of the trait are necessary for breeding and the trait utility within the selection process [23]. In Expt.1, higher heritability found in PH, RSA, RSR, LAI and RB indicated that the five traits were highly heritable, and strong positive correlation was observed among the five parameters under normal and drought conditions. There were significant differences in plant height, main stem node number, yield, and internode length in soybean between cultivars [1]. The Expt. 2 demonstrated that the significant differences were found for indicators such as PH, MSNN, PPP, SPP, HSW, YPP, RB, STB, and PB between variety treatments, and all these indicators presented higher *H*^*2*^ values. Therefore, the 9 secondary characteristics could be considered as criteria for drought resistance assessment.

Previous research has conclusively indicated positive correlations between MFVD and the drought-tolerant coefficient of some traits including plant height, grain number per spike, biological yield per plant, grain yield per plant, thousand kernel weight in wheat [23], which is also supported by our results; the conclusion stated clearly that MFVD was significantly positively correlated with the DC_RB_, DC_R/S_, DC_RSA_, DC_RSR_ and DC_RBR_ during vegetative growth period and DC_YPP_, DC_RB,_ and DC_PB_ during reproductive growth period. It is therefore concluded that the less the values of these traits decreased under drought stress, the more drought-tolerant the genotypes were. In addition, the DC of some traits were positively correlated with each other such as DC_PH_ and DC_LAI_, DC_PH_ and DC_SB_, DC_TRL_ and DC_RSA_, DC_PH_ and DC_MSNN_, DC_MSNN_ and DC_PPP_, DC_YPP_ and DC_SPP_. As compared with different drought criteria, we revealed that root-related indicators had relatively higher correlation with drought tolerance of plant than other ones tested, thus we regarded the root system as more valuable standard for evaluating the drought resistance of soybean. Similar studies have also pointed out the important role of roots in enhancing plant resistance to drought stress [50–52].

The drought resistance is a complex characteristic. A single characteristic cannot reflect the complex traits of the drought resistance mechanism, so more traits should be considered to evaluate the drought resistance in soybean. Stepwise multiple linear regression analyses revealed that the drought-tolerant coefficient of R/S, TRL, LAI and RSR could explain 73.70% of the total variation in MFVD at VGP, but higher heritability only found in LAI and RSR indicated that the 2 traits could serve as reliable indicators in drought evaluation. This again showed that the root system plays an important role in improving the drought resistance. Root traits contribute to drought avoidance of plants, which is relevant to avoid agricultural drought and sustain crop performance [53]. When soil moisture is insufficient, root traits are also essential for maintaining crop yields [54]. Many scientists have also claimed that modifying the root system structure will enhance crop yields and achieve a new green revolution [55, 56]. In addition, stepwise multiple linear regression analyses illustrated that the DC of SPP, YPP, PH, PB, MSNN and STB could explain 94.30% of the variation in MFVD at RGP. As a result, those six traits could be used as a combination to screen soybean genotypes for drought resistance.

## Conclusions

Even though drought-tolerant indices have many advantages, it is more and more widely used in combination with the value of membership function to screen drought resistant varieties in many crops. The present investigation was carried out to identify drought tolerance genotypes integrated the two methods which is needed for the development of soybean varieties in the arid and semi-arid areas. As a result, 3 accessions (LD24, JD36 and TF31) with drought tolerant during vegetative growth phase and 2 cultivars (TD37 and LD26) with highly drought resistant at reproductive growth stage were screened according to MFVD under WS and WW conditions, respectively. Meanwhile, based on DC and yield in the semi-arid zone, 50 genotypes were classified as drought tolerance and high yield (DD14, JD36, JD37, LD18, LD24, LD25, LD29, LD30, SN12, SN16, SN17, TF31, TD39, TD43, TD45, TD46, TD48, TD49, TD50, TD55, TD56, TD57, YW9), non-drought tolerance and high yield (LX2, TD47), drought tolerance and low yield (DD11, KY11, LD17, LD23, LD26, LD31, TD37), and non-drought tolerance and low yield (DD12, DD13, DD15, FD17, KY12, L08–28, LD15, LD21, LD22, LD28, SN8, SN10, SN11, TD38, TD40, TD42, XY11, YW6). In comparison WS, WW treatment increased the mean values of 7 characteristics but decreased that of the others at vegetative growth stage, and increased that of 8 traits but only decreased that of 1 trait at reproductive growth stage for 20 varieties. Correlation analysis showed that the MFVD was significantly and positively correlated with DC_RB_, DC_R/S_, DC_RSA_, DC_RLR_, DC_RSR_ and DC_RBR_ of vegetative growth phase and DC_YPP_ and DC_RB_ of reproductive growth stage. The results from ANOVA analysis showed that there were significant differences (*P* < 0.05) for 9 traits (PH, LAI, SB, R/S, RSA, RLR, RSR, RDR, RBR) between two water regimes, for 11 tested indicators (PH, LAI, SB, TRL, RSA, RV, RD, RLR, RSR, RVR, RDR) among 20 cultivars, for 5 properties (TRL, RV, RD, RVR, RDR) between the interactions when water was controlled at vegetative growth stage. While there was significant difference (*P* < 0.05) for only one trait (PH) between two water regimes, for all 9 investigated features (PH, MSNN, PPP, SPP, HSW, YPP, RB, STB, PB) among 20 cultivars, and for 5 indices (PH, MSNN, SPP, HSW, STB, STB) between the interactions when water was controlled at reproductive growth phase. The *H*^*2*^ for the investigated traits was estimated by genetic variance and phenotypic variance in two water treatments. However, the *H*^*2*^ of only one trait (PH) was higher than 0.9 and that of two traits (RSA, RSR) were more than 0.7 during vegetative growth stage, while that of the 9 investigated traits were all more than 0.7 during reproductive growth stage. This indicated that the variations of these traits were mostly due to genetic differences and they were highly heritable traits. There was a significant and positive linear relationship between WS and WW condition for the five traits (PH, LAI, SB, RSA and RSR) at vegetative growth stage and for all the agronomic traits at reproductive growth stage and the whole growth stage.

## Methods

### The pot experiments

A total of 20 soybean varieties from Liaoning Academy of Agricultural Science and Tieling Academy of Agricultural Science were selected in the present study to determine their drought tolerance properties at the vegetative growth stage (Expt. 1) and the reproductive growth stage (Expt. 2) under water stress conditions. The origin and description of 20 soybean varieties are shown in Table 12. The controlled experiments were conducted in a greenhouse of Liaoning Academy of Agricultural Science in Shenyang, Liaoning, China (41°49′ N, 123°32′ W). For both experiment seeds were sowed in plastic pots (28 cm average diameter and 28 cm average tall) contained 15 kg air dry brown soil from 0 to 20 cm plough layer. Soil samples were collected before planting to analyze the characteristics shown in Table 13. Six soybean seeds were sown in each pot and the population was thinned to two plants per pot when the first trifoliate leaf emerged. The pots were arranged in the electric movable greenhouse which was opened in the sunny day to maintain living conditions in a natural environment and closed in the rainy days to avoid the rain soaking plastic basin.

**Table 12:** The origin and description of 20 soybean studies varieties

Cultivar Name	Origin	Description
LD10	Shenyang, China	Commercial cultivar (LD3 × L82–5185)
LD15	Shenyang, China	Commercial cultivar (L85062 × ZCY18)
LD17	Shenyang, China	Commercial cultivar (LD3 × L92-2738 M)
LD18	Shenyang, China	Commercial cultivar (L89094 × L93040)
LD21	Shenyang, China	Commercial cultivar (L8878× L93009)
LD23	Shenyang, China	Commercial cultivar (LD10 × L91086)
LD24	Shenyang, China	Commercial cultivar (LD3 × YPZ)
LD26	Shenyang, China	Commercial cultivar (L8880 × IOA22)
JD36	Jinzhou, China	Commercial cultivar (JD2 × TF18)
JD37	Jinzhou, China	Commercial cultivar (MC25 × L9825)
KY11	Kaiyuan, China	Commercial cultivar (KJ7528× GZM)
KY12	Kaiyuan, China	Commercial cultivar (K8525 × KJ8157)
TF29	Tieling, China	Commercial cultivar (8114 × 84059)
TF31	Tieling, China	Commercial cultivar (LD3 × Resnick)
TD37	Tieling, China	Commercial cultivar (89034–10 × TF29)
TD40	Tieling, China	Commercial cultivar (89078 × 92035)
TD49	Tieling, China	Commercial cultivar (93058–19 × TF29)
DD12	Dandong, China	Commercial cultivar (D806 × LD10)
SN10	Shenyang, China	Commercial cultivar (SN92–16 × SN91–44)
FD17	Fushun, China	Commercial cultivar (F82–47× DJ1)

**Table 13:** Some initial properties of the soils in each experiment

Experiment number	Location	pH	OM (%)	Total N(%)	Total P(%)	Total K(%)	Avai.N (mg kg^−1^)	Avai.P (mg kg^−1^)	Avai.K (mg kg^− 1^)
Expts. 1, 2	Shenyang	6.90	1.18	0.12	0.08	2.42	100	16.8	98
Expt. 3 (2014)	Shenyang	6.60	2.68	0.12	0.18	2.64	100	25.4	135
	Chaoyang	6.90	2.12	0.11	0.16	2.78	94.0	22.8	164
Expt. 3 (2015)	Shenyang	6.60	2.58	0.13	0.17	2.58	110	24.8	148
	Chaoyang	6.90	2.24	0.11	0.17	2.75	102	23.4	175

Two series of pot experiments were arranged in two factors randomized complete block design with three replicates. The water treatments included well-watered (WW) and water-stressed (WS) regimes. The soil water content of WW regime was maintained at field capacity, and that of WS regime was 50% of the field capacity. Twenty domestic soybean genotypes used in the experiments. Two pots for each genotype with two water regimes were grown next to each other in pairs. For each pair the treatments were randomly assigned to each pot. The soil water content and field capacity were determined before the experiment, and then calculate the weight of pot and soil for each water treatment (plant weight is ignored). The pots were weighed every 2 d to maintain soil moisture at the target weight by rewatering.

#### Expt. 1

Water-stressed (WS) was applied 30 days when the plants had three fully expanded leaves and ended at flowering phase. Thereafter, plants were harvested and a total of 14 traits were investigated and calculated. Definition of traits and their description of measurement are listed in Table 14.

**Table 14:** Trait name and description of their measurement and calculation

Experiment No.	Trait names and description of their measurement and calculation
Expt. 1	Plant height (PH, cm), the average height of the two individuals per pot from cotyledonary node to the main stem top. Leaf area index (LAI), total leaf area per unit ground area, and leaf area was measured with a leaf area meter (LI-3100C, LI-COR, Lincoln, NE, USA). **Shoot biomass (SB, g plant**^**−1**^**) and root biomass (RB, g plant**^**−1**^**),** the average of above ground dry weight and that of root dry weight of the two plants per pot after drying to constant weight at 80 °C in a drying oven, respectively**.** Root/shoot ratio (R/S), calculated as root dry weight/shoot dry weight**;** Total root length (TRL, m), root surface area (RSA, cm^2^), root average diameter (RD, mm) and root volume (RV, cm^3^), calculated on the average of two individuals per pot and measured using WinRHIZO (EPSON 1680, WinRHIZO Pro2003b, Regent Instruments Inc., Quebec, Canada). Root length ratio (RLR, m/g), total root length/total biomass. Root surface area ratio (RSR, cm^2^/g), root surface area/total biomass; Root volume ratio (RVR, cm^3^/g), root volume/total biomass; root average diameter ratio (RDR, mm/g), root diameter/total biomass; root biomass ratio (RBR), root biomass/total biomass.
Expt. 2	Plant height (PH, cm), the average height of the two individuals per pot from cotyledonary node to the main stem top. Main stem node number (MSNN), Pods per plant (PPP) and Seeds per plant (SPP), measured on the average of 2 plants in the pot at maturity; Hundred seeds weight (HSW, g), calculated the weight of 100 seeds; Yield per plant (YPP, g **plant**^**−1**^), the average yield of the two individuals per pot. Root biomass (RB, g **plant**^**− 1**^), Stem biomass (STB, g **plant**^**− 1**^) and Pod biomass (PB, g **plant**^**− 1**^), measured on the average of 2 plants which were separated into bean, root, stem and pod determined after drying, respectively.
Expt. 3	Plant height (PH, cm), First pod height (FPH, cm), Main stem node number (MSNN) and Branches (BR), were estimated in 5 plants per plot.

#### Expt. 2

Water-stressed (WS) was applied 50 days at the beginning of flowering R1 until the first physiological maturity pod appearance R7. After all plants maturity, plants were harvested and a total of 9 traits were investigated which are listed in Table 14.

#### Expt. 3 (field studies)

Fifty soybean genotypes from Liaoning Academy of Agricultural Science and Tieling Academy of Agricultural Science including 18 varieties in Expts. 1 and 2 were evaluated for drought tolerance in Chaoyang (41°30′ N, 120°29′ W, 170 m a.s.l.) located in the semi-arid zone and Shenyang (41°82′N, 123°55′W, 52.9 m a.s.l.) located at humid and sub-humid region, representing different rainfall characteristics in 2014–2015. Both Chaoyang and Shenyang are temperate continental monsoon climate type with severe and dry winter, and high temperature and concentrated rainfall in summer. The annual average sunshine, the mean temperature, and frost free days in a normal year in Chaoyang are around 2900 h, 6 °C and 135 d, respectively, and those in Shenyang are around 2400 h, 8 °C and 150 d, respectively.

The field experiment was arranged in complete block design replicated three times with two locations (Shenyang representing well-watered condition is located in a humid and semi-humid area; Chaoyang representing water stress condition is located in a semi-arid area). Both Chaoyang and Shenyang, 50 genotypes were sown in field plots, and each plot consisted of four 6 m rows, in East-West orientation, with 0.60 m inter-row spacing. Plots were over-seeded with hand planters and seedlings thinned to a final stand of 166,700 plants ha^− 1^. The plots were fertilized with 45–70-60 kg ha^− 1^ in the form of N-P_2_O_5_-K_2_O before planting. The soil samples of two locations were typically brown soil that was taken before planting to analyze the characteristics given in Table 13.

After maturity, an area of 6.0 m^2^ was harvested by hand from the two central rows from each plot. The whole harvested area was used to determine yield. Five plants from the second row were harvested and a total of 4 traits were investigated which were listed in Table 14.

### Estimation of drought-tolerant coefficient (DC) and membership function value of drought tolerance (MFVD)

The drought tolerance coefficient (DC) is calculated according to the following equation: data ratio derived from the WS and WW regimes of the same genotype for each trait [23, 57, 58].

$$ DC{}_{ij r}={T}_{ij wsr}/{T}_{ij wwr}\kern0.5em {DC}_{ij}=\frac{1}{r}\sum \limits_{ij=1}^r DC{}_{ij r} $$

where *DC*_*ijr*_ is the drought-tolerant coefficient of the *j-th* trait for the *i-th* cultivar in the *r-th* replication; *T*_*ijwsr*_ and *T*_*ijwwr*_ are the value of the *j-th* trait for the *i-th* cultivar evaluated under WS and WW treatments in the *r-th* replication, respectively; *DC*_*ij*_ is the average value of drought-tolerant coefficient of *j-th* trait for the *i-th* cultivar.

Soybean drought tolerance was also evaluated by the membership function value. This methodology gives a comprehensive assessment by using the membership functions based on the theory of fuzzy mathematics. The membership function of a fuzzy set is a generalization of the indicator function in classical sets; it represents the degree of truth as an extension of valuation [59]. For any set *T*, a membership function on *T* is any function from *T* to the real unit interval [0,1]. According to the DC, the modified MFVD was calculated following the equations:

$$ {F}_{ij}=\frac{DC_{ij}-{DC}_{jmin}}{DC_{jmax}-{DC}_{jmin}}\kern0.5em {F}_i=\frac{1}{n}\sum \limits_{j=1}^n{F}_{ij} $$

where *F*_*ij*_ is the membership function value of the *j-th* trait for *i-th* cultivar for drought tolerance; *DC*_*jmax*_ and *DC*_*jmin*_ were the maximum value and minimum value of the drought resistant coefficient for the *j-th* trait, respectively; *F*_*i*_ is the average value of the membership function of measured traits for the *i-th* cultivar for drought tolerance.

Drought tolerance is divided into five levels according to the average value ($\overline{F_i}$) and standard deviation (*SD*) of MFVD in two series of pot experiments. Class and level to drought resistance are listed in Table 15.

**Table 15:** Class and level to drought resistance of soybean genotypes according to the $\overline{F_i}$ and *SD* of MFVD

Level	*F*_*i*_	Class
**1**	*F*_*i*_ ≥ $\overline{F_i}$ + 1.64*SD*	Highly drought tolerant
2	$\overline{F_i}$+ 1.64*SD* > *F*_*i*_ ≥ $\overline{F_i}$ + *SD*	Drought tolerant
**3**	$\overline{F_i}$+ *SD* > *F*_*i*_ ≥ $\overline{F_i}$- *SD*,	Moderate drought tolerant
**4**	$\overline{F_i}$- *SD* > *F*_*i*_ ≥ $\overline{F_i}$- 1.64*SD*	Susceptible
5	*F*_*i*_ < $\overline{F_i}$- 1.64*SD*	Highly susceptible

Evaluation and classification of drought tolerance of soybean genotypes at the whole growth stage based on yield relative drought index (RDI) and yield in semi-arid areas in the field experiment. RDI of yield was calculated as [60]:

$$ RDI=\frac{Y_{iws}}{Y_{iww}}\div \frac{Y_{mws}}{Y_{mww}} $$

where *Y*_*iws*_ and *Y*_*iww*_ were the yield of tested *i-th* genotype and *Y*_*mws*_ and *Y*_*mws*_ were the mean yield of all genotypes under water-stressed (semi-arid region) and well-watered (humid and sub-humid region) conditions, respectively.

### Data analysis

Multiple linear regression was performed with SAS 8.0 statistical software to construct the select indices of MFVD using multiple DC of some traits. Analysis of variance (ANOVA), correlation and heritability (*H*^*2*^) analyses were carried out for all the data sets using SPSS 16.0 statistical software, and significance differences was determined at the significances of 0.05, 0.01, 0.001 probability level using the Duncan’s tests.

Variation was partitioned into relevant sources of variation to test for differences among genotypes. The linear model was used as follows:

$$ {Y}_{rki}=\mu +{R}_r+{W}_k++{G}_i+{WG}_{ki}+{\varepsilon}_{rki} $$

where *Y*_*rki*_: observation corresponding to *kth* level of water factor, the *ith* level of genotypic factor and the *rth* replication; *μ*: general mean; *R*_*r*_: the *rth* replication effect; *W*_*k*_: the *kth* level of water treatment effect; *G*_*i*_: the *ith* genotypic treatment effect; *WG*_*ki*_: interaction between the *ith* level of genotypic treatment and the *kth* level of water treatment; and *ε*_*rki*_: the residual error.

The genetic variation coefficient (*CV*_g_) of each trait was calculated following the equation:

$$ {CV}_g=\frac{SD}{\overline{X}}\times 100\% $$

where *SD* is standard deviation, $\overline{X}$ is the average value of the trait under the same water-controlled conditions.

The broad sense heritability (*H*^*2*^) of each trait was calculated following the equation:

$$ {H}^2=\frac{V_g}{V_g+{V}_{wg}+{V}_e} $$

Where *V*_g_ is genotypic variance, *V*_*wg*_ is the interaction variance between genotype and water treatment, *V*_*e*_ is error variance.

## Data Availability

All datasets generated or analyzed during this study are available from the corresponding author on reasonable request.

## Abbreviations

WS: Water-stressed
WW: Well-watered
MFVD: Membership function value of drought tolerance
DC: Drought-tolerant coefficient
VGP: Vegetative growth phase
RGP: Reproductive growth phase
*H*^*2*^: Broad sense heritability
OM: Organic matter
Avai.: Available
N: Nitrogen
P: Phosphorus
K: Potassium
DC_PH_: Drought-tolerant coefficient of plant height
DC_LAI_: Drought-tolerant coefficient of leaf area index
DC_SB_: Drought-tolerant coefficient of shoot biomass
DC_RB_: Drought-tolerant coefficient of root biomass
DC_R/S_: Drought-tolerant coefficient of root/shoot ratio
DC_TRL_: Drought-tolerant coefficient of total root length
DC_RSA_: Drought-tolerant coefficient of root surface area
DC_RV_: Drought-tolerant coefficient of root volume
DC_RD_: Drought-tolerant coefficient of root average diameter
DC_RLR_: Drought-tolerant coefficient of root length ratio
DC_RSR_: Drought-tolerant coefficient of root surface area ratio
DC_RVR_: Drought-tolerant coefficient of root volume ratio
DC_RDR_: Drought-tolerant coefficient of root average diameter ratio
DC_RBR_: Drought-tolerant coefficient of root biomass ratio
DC_MSNN_: Drought-tolerant coefficient of main stem node number
DC_SPP_: Drought-tolerant coefficient of seeds per plant
DC_PPP_: Drought-tolerant coefficient of pods per plant
DC_HSW_: Drought-tolerant coefficient of hundred seeds weight
DC_STB_: Drought-tolerant coefficient of stem biomass
DC_PB_: Drought-tolerant coefficient of pod biomass
DC_YPP_: Drought-tolerant coefficient of yield per plant
